# Salivation Caused by Fillings of the "Amalgam of Mercury and Silver" in the Teeth

**Published:** 1842-06

**Authors:** 


*Salivation caused by fillings of the "Amalgum of Mercury and Silver" in the Teeth.*—We have before had occasion to speak of the hurtful effects that result from the employment of the "amalgum of mercury and silver," for filling teeth. We could mention many cases where its use has been followed by serious consequences, and that ptyalism is often, in this way, produced, is a fact, now, too well established to admit of doubt. A case was related to us a few days since, of a gentleman, a resident of the District of Columbia, who, about eighteen months since, while in the city of London, applied to a dentist for the purpose of having several large cavities in his teeth, filled. The operation was performed in a few minutes, and to the gentleman's great satisfaction. In a few days, however, his gums began to inflame and swell, and these symptoms were gradually followed by an increased flow of saliva, inflammation of the mucous membrane of the mouth generally, and of the alveolo-dental periostea, soreness and loosening of all his teeth, fetor of the breath, anorexy and all the other symptoms attendant upon a mercurial diathesis of the system. Having visited Paris about three weeks after the operation, and the symptoms becoming more and more aggravated every week, he at length consulted an eminent medical gentleman of that metropolis, who, after having carefully investigated his case, informed him that he was salivated. But when the gentleman assured him that he had not taken mercury in any form for ten years, and that he had never been salivated in his life, his physician stated that if the opinion which he had given was incorrect, he was ignorant of the nature of his disease. Learning, however, in the course of the conference, that he had had a number of his teeth filled, ten or twelve weeks before, in London, with a "mineral paste," he was induced to examine the fillings, which proving to be an amalgum of mercury and silver, the mystery was at once explained. The fillings, of course, were at once removed, and though the constitutional symptoms, as we were informed, soon subsided, there still remains a chronic inflammation and thickening of the alveolo-dental membranes and looseness of the teeth.

